# Genito-Urinary and Syphilitic Diseases

**Published:** 1901-08

**Authors:** Byron H. Daggett

**Affiliations:** Buffalo, N.Y.


### Genito-Urinary and Syphilitic Diseases.

Conducted by BYRON H. DAGGETT, M. D., Buffalo, N. Y.

### THE ETIOLOGY AND RATIONAL TREATMENT OF RENAL DISEASES, COMMONLY CLASSED AS "BRIGHT'S DISEASE."

Porter, W. H., (*Post Graduate*, December, 1900,) states that in order to understand the etiology and treatment of these renal diseases, the physician must have a clear knowledge of the anatomy, physiology, as well as the pathology and symptomatology of these glands.

Histologically, these organs are composed of two distinct kinds of tissues, viz.: the epithelial and connective tissue groups. The latter is the foundation or base for the epithelia and the intervening tissue about the tubules, vessels and nerves. All of these make up the intertubular structure. The vascular portion —particularly the malpighian tufts—secrete the water with the inorganic salts, while the intertubular portion of the vascular areas bring the proteids and oxygen into close relation with the uriniferous tubules, when the protoplasmic cells reduce them into catabolic excreta, such as urea, uric acid, kreatin, water and carbon-dioxide. The carbon dioxide is taken up by the alkaline blood and eliminated by the lungs. The water eliminated by the malpighian tufts is the water taken into the system under its own form, but the water which enters through the epithelial cell is formed by the oxidation of the proteid molecule. So long as the renal gland is not overtaxed and the nutrition supply is of the right kind and gravity, the organ will physiologically perform its work. Overload this gland or impair its nutrition and innervation and retrograde changes will follow and cause renal affections, classed as Bright's disease.

In the event that there be an insufficient supply of oxygen, then there will be imperfect oxidation reduction and portion of the proteid molecules will be isomerically transformed in the protoplasm in the cells of the renal tubules, and eliminated as such into the lumen of the uriniferous tubules. In the milder forms of incomplete oxidation of the proteid elements, there is a lessened output of urea and an increased output of uric acid. In the more aggravated conditions various products indicative of still more incomplete oxidation reduction are eliminated, such as oxalic acid, lactic acid, and the like. The proteid molecules are isomerically transformed in the absence of oxidation and eliminated, as nucleo-albumin. They are eliminated as isomeric forms of albumin, as found in the urine. A careful examination of the urine will determine the ability, the perfection or imperfection of the system to transform the proteids.

The writer gives a method for detecting the reduction or lack of reduction of the proteid substances. This method is described as follows: Fill a test tube nearly full of urine, bring the upper stratum to the boiling point, then add a few drops of 4 per cent.

acetic acid solution, and again bring to the boiling point, then set aside for a few hours to cool. If the excretion of uric acid is normal in amount no formation of uric acid crystals will be found in the urine examined. If, on the other hand, there is an abnormal excretion of uric acid that is held in solution, the heat and the acetic acid starts up a chemical action by which the acid phosphate of soda is formed, and the uric acid precipitated. The crystals of uric acid begin to form directly under the surface layer of the urine. The amount formed will give the relative quantity excreted. While this is not an absolute quantitative method of estimating the excess of uric acid, it is one that is practical and tells us very quickly that there is an over-production, and also the relative amount of the over-production from day to day.

The uric acid may be precipitated and weighed and thus get the absolute quantity of free acid. The strong mineral acids, however, decompose the uric acid salts, and you get the total output of uric acid.

There are two forms of anatomical changes in these glands, viz.: one implicating the epithelial structures; the other affects the intertubular portion. The one is a parenchymatous, the other is an interstitial lesion. These two forms may combine, making a diffuse lesion. Intense congestion and blood toxicity incite an exudative or diffuse nephritis. The other forms are not inflammatory.

Causes of Bright's disease:

1. Taking into the system more proteids than are required for nutrition and growth; surcharging the blood and overworking the kidneys in their elimination. The transitory presence of albumin in the urine does not necessarily indicate a renal lesion; it may indicate elimination of a surplus, which threatens the integrity of the renal glands.

2. Another cause of degenerative changes in the renal epithelia is due to the elimination of the products of intestinal putrefactive changes. They are toxic and tend toward degeneration of the kidney in its efforts to transform and eliminate.

Indican is formed by the putrefaction of proteids in the intestinal canal. Indican uniting with a potassium salt forms indoxyl of sulphate of potassium, which obeys the law of inorganic substances, is readily absorbed into the circulation and eliminated by the renal glands. Its appearance in the urine is a danger signal, warning the physician of impending danger to renal structures.

Jaffe gave the following method for the detection of this substance: Take equal volumes of chemical hydrochloric acid and urine, add a few drops of a ½ per cent. of potassium permanganate. If indican is present there will be a blue discoloration of the fluid. A small quantity of chloroform will cause a blue deposit and the amount and the intensity of the deposit will indicate a large or small amount of the indoxyl of sulphate of potassium.

3. Microörganisms are the most powerful factors in causing degeneration of renal cells. They act directly within the system and indirectly upon the alimentary contents. They produce toxics and the oxidation reduction products or the isomeric proteid elements. They impair nutrition, overburden the kidneys and beget degeneration.

Oxidation reduction proteids are found in the cells of glands and not in the blood or lymph, and it seems probable that they are isomeric.

Adami (Montreal) affirms that renal lesions are the result of the direct action of bacterial products, and that the bacterial action is within the system. It is more probable, however, from the evidence at our command, that the bacterial action, producing renal lesion, is brought about in the alimentary canal and not within the system.

The interstitial form of Bright's disease is largely dependent upon these conditions which cause the parenchymatous changes. The phenomenon and the etiological explanation of the development of the interstitial forms of renal disease is complicated and extensive, so that abstraction is not practical.

With a good understanding of the causes of renal lesions, their treatment is governed by a few plain and common principles. Knowing that the parenchymatous lesions are caused by over-eating and over-drinking, it is apparent that a dietary must be prescribed, and the intestinal canal freed from putrefaction and its results. The quantity of the food must be kept within the oxygenating capacity of the economy, to save the renal glands from overwork and faulty nutrition. Putrefactive fermentation in the alimentary canal is quickly manifested in the urine by varying quantities of indican.

Uric acid in excess of the normal quantity indicates overfeeding or imperfect oxidation, from over abundance of food or deficient amount of oxygen. Too much stress cannot be laid upon the significance of these two substances in the urine, nor upon the importance of recognising their presence in the urine early, and also the necessity of removing the conditions that make possible their presence in the urine, if prevention or cure of renal lesions is to be successfully accomplished. Both of these conditions are positive indicators of a deteriorated state of the animal economy, and one which tends to lower the general nutritive activity of the system and invite bacterial invasion, for without a suitable medium the germs cannot thrive in the economy, nor produce their damaging effects. Therefore it becomes clear that to prevent an invasion of the system by bacteria, whose poison will eventually damage the renal structures, the two conditions just enumerated must be prevented.

